# Méningo-encéphalite à *Haemophilus influenzae* type b chez le grand enfant: à propos d’un cas

**DOI:** 10.11604/pamj.2022.42.220.35804

**Published:** 2022-07-20

**Authors:** Ines Trabelsi, Mouadh Ben Ali, Manel Ben Romdhane, Hanen Smaoui, Imen Bel Hadj, Khadija Boussetta

**Affiliations:** 1 Service de Médecine Infantile B, Hôpital d’Enfants « Béchir Hamza » de Tunis, Tunis, Tunisie,; 2 Service de Microbiologie, Hôpital d´Enfants de Tunis, Tunis, Tunisie

**Keywords:** Méningo-encéphalite, enfant, *Haemophilus influenzae* type b, vaccination, cas clinique, Meningoencephalitis, child, Haemophilus influenzae type b, vaccination, case report

## Abstract

Les infections invasives à Haemophilus influenzae de type b (Hib) sont devenues rares après la généralisation de la vaccination. Nous rapportons le cas d´un garçon âgé de 9 ans admis dans un tableau d´état de mal convulsif dans un contexte de fièvre avec altération de l´état de conscience. L´examen initial avait trouvé un enfant comateux, score de Glasgow à 9/15, une fièvre à 38,2, des réflexes ostéo-tendineux vifs sans syndrome méningé franc. Le bilan biologique avait montré une hyperleucocytose à polynucléaires neutrophiles (PNN) avec une CRP à 45,8. L´étude du liquide céphalorachidien avait montré un aspect trouble, une pléiocytose à 6760 éléments blancs/mm^3^ avec une prédominance neutrophile (PNN = 90%, Lymphocytes = 10%). L´examen direct a montré des bacilles polymorphes, l´antigène soluble était positif à l´Haemophilus influenzae d type b. La glycorachie était effondrée à 0.04 mmol/Let la protéinorachie était franchement élevée à 4.097 g/L. L´imagerie cérébro-médullaire par résonance magnétique a montré un aspect évocateur d´encéphalite de topographie sous et sus tentorielle avec des anomalies de signal cortico-sous corticales pariéto-occipitales et cérébelleuses bilatérales. L´évolution était favorable sous Céfotaxime. Devant ce tableau d´infection invasive à Hib, la vérification du carnet de santé de notre patient a montré une absence de vaccination contre le Hib durant la petite enfance. Après un recul de 3 ans, il est asymptomatique ne présentant pas de séquelles neurosensorielles. Le développement d´une infection sévère à Hib doit faire obligatoirement vérifier la réalité de la vaccination ou rechercher un déficit immunitaire sous-jacent.

## Abstract (en)

Since widespread vaccination, invasive Haemophilus influenzae type b (Hib) has become a rare infection. We here report the case of a 9-year-old boy admitted with seizures associated with fever and impaired general condition. First examination showed comatose child, Glasgow score 9/15, fever 38.2, deep tendon reflexes without frank meningeal syndrome. Laboratory tests showed polymorphonuclear neutrophils (PNN) with CRP 45.8. Cerebrospinal fluid (CSF) analysis revealed a cloudy appearance, pleiocytosis (6760 white blood cell count/ mm^3^) with neutrophil predominance (PNN = 90%, lymphocytes = 10%). Direct examination showed polymorphic bacilli, soluble antigen of Haemophilus influenzae type b, decreased glycorachy 0.04 mmol/L and hyper proteinorachie 4.097 g/L. MRI of the cerebellomedullary fissure revealed subtentorial and supratentorial encephalitis with bilateral parieto-occipital and cerebellar cortical and subcortical signal anomalies. The patient was treated with cefotaxime with favorable outcome. The patient had not been vaccinated against Hib in early childhood. After a 3-year follow-up, the patient was asymptomatic with no neurosensory sequelae. In subjects with severe Hib infection proof of vaccination or testing for underlying immunodeficiency are required.

## Introduction

Les infections à *Haemophilus influenzae* sont associées à une morbidité importante et une mortalité non négligeable. L *´Haemophilus influenzae* est responsable d'infections communautaires de la sphère otorhinolaryngologique et de surinfections broncho-pulmonaires chez l´enfant et chez l´adulte. Ces infections sont généralement provoquées par des souches non capsulées. Parmi les souches encapsulées, l´*Haemophilus influenzae* de sérotype b (Hib) est le plus pathogène puisqu´il est responsable de plus de 90% des infections invasives graves à l´*Haemophilus influenzae* chez l´enfant, principalement chez les enfants de moins de 5 ans [1]. Les deux principales infections graves à Hib sont la méningite et la pneumonie. D´autres localisations sont décrites: l´épiglottite, la septicémie, l´arthrite septique, l´ostéomyélite, la cellulite et la péricardite [1,2]. A notre connaissance, la méningoencéphalite à l´*Haemophilus influenzae* était peu rapportée dans la littérature depuis la généralisation de la vaccination. Nous rapportons le cas d´une méningite à Hib chez un grand enfant admis dans un tableau de méningoencéphalite en soulignant la rareté de l'*Haemophilus influenzae* dans les méningites bactériennes chez l´enfant et en insistant sur les difficultés diagnostiques rencontrées.

## Patient et observation

**Informations du patient:** MN un enfant de 9 ans, de sexe masculin, a été hospitalisé pour des convulsions dans un contexte fébrile. Il n´avait pas d´antécédents familiaux de convulsions fébriles ou d´épilepsie. Il était né à terme par voie basse avec une bonne adaptation à la vie extra-utérine. Il n´avait pas d´autres antécédents pathologiques notables. Il avait une bonne croissance staturo-pondérale et un bon développement psychomoteur. Il n´avait pas d´histoire de traumatisme récent ni de contage viral ni d´une ingestion de plante inconnue ou une exposition aux toxiques. L´histoire de la maladie remontait à 3 jours avant l´admission où l´enfant avait présenté des céphalées associées à une rhinorrhée et une fièvre oscillant entre 38 et 38,5°C, sans diarrhée ni vomissements ni toux. Le jour de l´admission, notre patient a présenté des vomissements suivis d´un état de mal convulsif avec des crises de 5 à 10 minutes entrecoupées de périodes d´accalmies de 5 à 10 minutes, sans reprise de la conscience.

**Résultats cliniques:** MN a été hospitalisé 160 minutes après le début des convulsions. L´examen initial a montré une fièvre à 38.2°C, une glycémie capillaire correcte à 1.19 g/L. MN était comateux avec un score de Glasgow à 9/15; il n´était pas en train de convulser. On n´a pas retrouvé un syndrome méningé franc. Les pupilles étaient initialement en mydriase réflective. Les réflexes ostéo-tendineux étaient présents, symétriques mais vifs. L´état hémodynamique était stable avec une pression artérielle de 12/7 (normale pour la taille), une fréquence cardiaque à 90 bpm, une auscultation cardiaque normale, un temps de recoloration cutané immédiat et des pouls périphériques présents et symétriques. MN était rose avec une SpO_2_=98% à l´air ambiant, il était eupnéique avec une fréquence respiratoire à 18 cpm, l´auscultation pulmonaire était sans anomalies. L´abdomen était souple indolore sans masse palpable. L´examen cutané était normal, il n´y avait pas de purpura ni de tâches café au lait ni de tâches achromiques. Les bandelettes urinaires n´ont pas retrouvé ni hématurie ni protéinurie ni leucocyturie ni acétonurie ni glycosurie. Au total il s´agit d´un garçon de 9 ans ayant présenté un état de mal convulsif dans un contexte de fièvre avec altération de l´état de conscience.

**Démarche diagnostique:** la tomodensitométrie initiale n´a pas montré d´anomalies. Le bilan biologique avait montré une hyperleucocytose à prédominance neutrophile (GB=22520/mm^3^, PNN = 20450/mm^3^), une lymphopénie à 640/mm^3^et une monocytose à 1410/mm^3^. L´hémoglobine était à 11.8 g/dL et le taux de plaquettes était à 199 000/mm^3^. La Creactive protein (CRP) était à 45.8 mg/L. L´ionogramme sanguin, le bilan rénal, le bilan hépatique et la calcémie étaient normaux. La recherche de toxiques (salicylés, barbituriques, benzodiazépines, carbamazépines, opiacés) dans le sang et les urines était négative. L´étude du liquide céphalorachidien a montré un aspect trouble, une pléiocytose à 6760 éléments blancs/ mm^3^ avec une prédominance neutrophile (PNN = 90%, Lymphocytes = 10%). L´examen direct a montré des bacilles polymorphes, l´antigène soluble positif à Hib. La glycorachie était effondrée à 0.04 mmol/Let la protéinorachie était franchement élevée à 4.097 g/L.

**Figure 1:**
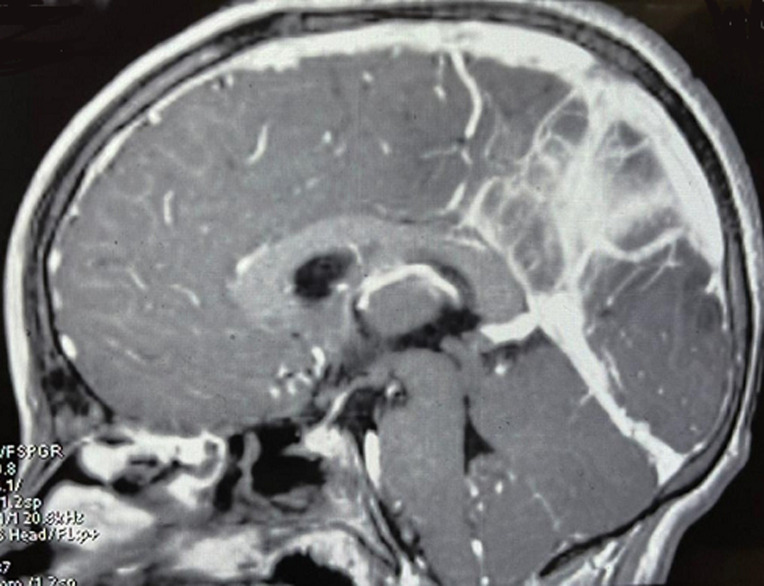
coupe sagittale de l´IRM cérébrale montrant des anomalies de signal cortico-sous-corticales, pariéto-occipitales bilatérales

**Intervention thérapeutique:** la conduite à tenir initiale était de mettre l´enfant sous Céfotaxime 200 mg/Kg/j en 4 prises, Dexaméthasone à la dose de 0.6 mg/kg/jour, Valproate de sodium à la dose de 30 mg/kg/jour. Devant ce tableau atypique de méningoencéphalite purulente à Hib chez le grand enfant, MN était également mis sous Aciclovir 500mg/ m^2^) SC/8h devant la suspicion d´une coïnfection herpétique en attendant le résultat de la recherche virale. La culture du liquide céphalo-rachidien a confirmé le diagnostic de méningite purulente en mettant en évidence un Hib. L´imagerie cérébro-médullaire par résonance magnétique, faite au bout de 48 heures d´hospitalisation, a montré un aspect évocateur d´encéphalite de topographie sous et sus tentorielle avec des anomalies de signal cortico-sous corticales pariéto-occipitales et cérébelleuses bilatérales en discret hyper signal T2 et Flair, hyper signal intense en diffusion sans prise de contraste après injection (Figure 1).

**Suivi:** l´évolution clinique était progressivement favorable avec obtention d´une apyrexie durable au 4^è^ jour d´antibiothérapie. Une amélioration de l´état de conscience a été observée à partir du 3^è^ jour de prise en charge. MN n´a pas reconvulsé. La recherche des virus Herpès simplex 1 et 2 (HSV1, HSV2) et entérovirus dans le liquide céphalorachidien par PCR était négative motivant l´arrêt de l´Aciclovir. En vérifiant le carnet de santé de notre patient: Il a été correctement vacciné, selon le calendrier national tunisien de vaccination, contre la tuberculose, l´hépatite virale B, la diphtérie, le tétanos, la coqueluche, la poliomyélite, la rubéole et la rougeole. Néanmoins, il n´a pas reçu la vaccination contre Hib en 2009-2010, ni contre le pneumocoque. Sur le plan biologique, la CRP a nettement augmenté avec un maximum à 292 mg/l au bout de 48 heures d´hospitalisation. Le bilan biologique de contrôle au 9^e^ jour de prise en charge était normal. MN a reçu le céfotaxime pendant 10 jours et la dexaméthasone pendant cinq jours. Il a été mis sortant au bout de 11 jours d´hospitalisation sous Valproate de Sodium. L´audiogramme n´a pas objectivé de surdité. Le traitement anticonvulsivant a été arrêté au bout de 5 mois devant l´absence de récidive et un aspect normal à l´électroencéphalogramme. Le recul actuel est de trois ans, MN n´a pas présenté de récidive de convulsion, n´a pas présenté de fléchissement scolaire.

## Discussion

La découverte de l´*Haemophilus influenzae* remonte à 1883 quand il a été mis en évidence pour la première fois par Koch dans un pus de conjonctivite, puis en 1893 par Pfeiffer dans les expectorations de malades atteints de la grippe [3]. L´*Haemophilus influenzae* est une espèce classée dans le genre *Haemophilus* au sein de la famille des *Pasteurellaceae*. Il se présente sous la forme de coccobacilles à Gram négatif. Certaines souches sont entourées d´une capsule polysaccharidique. Les souches encapsulées sont classées en six sérotypes (a, b, c, d, e, f) selon les caractéristiques immunogéniques de leur capsule. La capsule confère à ces sérotypes une virulence accrue car elle faciliterait la pénétration de la bactérie dans le sang provoquant ainsi des infections invasives plus graves notamment une septicémie ou méningite. La majorité des infections systémiques à *Haemophilus influenzae* est due aux souches capsulées de type b en raison du rôle majeur du polyribosephosphate (PRP) comme facteur de virulence qui confère une résistance à la phagocytose et à l'action du complément. Les autres sérotypes sont, en effet, éliminés au cours de la phase septicémique et présentent un risque moindre de localisations secondaires (arthrites, méningites) [4].

La vaccination contre l´*Haemophilus influenzae* a commencé en 1970. Elle a permis la régression de la prévalence des manifestations invasives. Depuis l´introduction de la vaccination aux USA, l´incidence des infections invasive à Hib est passée de 46-100 cas / 100000 enfants de moins de cinq ans à 1-4 cas / 100000 [5]. En Tunisie, le vaccin Hib était introduit en 2002 et retiré en 2005. Ce retrait était dû à une limitation budgétaire. Sa réintroduction en 2011 était sous forme d´un vaccin pentavalent [6]. Ce retrait explique l´absence de vaccination contre l´Hib de notre patient qui aurait dû être vacciné en 2009. En Tunisie, à l´instar de nombreux pays, les profils épidémiologiques et bactériologiques des méningites bactériennes se sont modifiés en raison de l´introduction de la vaccination obligatoire contre le Hib en 2011 dans le calendrier vaccinal national. En effet, une étude hospitalière menée dans le sud Tunisien, avant 2011, a colligé 12 cas (44%) de méningite purulente à Hib sur 27 cas confirmés [7]. Une diminution du nombre de méningites purulentes à Hib était notée dans l´étude de Tfifha *et al*., menée entre 2006 et 2016, passant de huit cas avant 2011 à trois cas à partir de 2011 [8]. L´Hib peut être rarement responsable d´atteinte encéphalitique soit primitive soit secondaire à une infection du système nerveux central (une méningite, un abcès cérébral ou une thrombophlébite cérébrale). L´encéphalite peut être soit réplicative, comme chez notre patient, soit non réplicative post infectieuse [9].

Le tableau clinico-biologique de la méningoencéphalite à Hib est trompeur avec un tableau encéphalitique fluctuant, une fièvre modérée, un syndrome méningé fonctionnel et physique peu évident et un syndrome inflammatoire biologique modéré. Les méningoencéphalites d´origine virale peuvent donner un tableau similaire. L´examen chimique, bactériologique et virologique du liquide céphalo rachidien et l´imagerie cérébrale par résonnance magnétique permettent généralement d´orienter le diagnostic étiologique des méningoencéphalites. Une étude russe s´intéressant aux lésions du système nerveux central secondaires à l´Hib chez l´adulte a montré qu´une coinfection virale était présente dans presque la moitié des cas (7 patients sur 13) [10]. Etant donné la gravité de la pathologie et le risque des séquelles neurosensorielles qui peut être majoré par un retard diagnostique ou thérapeutique, il ne faut pas hésiter à débuter le traitement antibiotique et/ou le traitent antiviral dès la suspicion du diagnostic et adapter secondairement le traitement en fonction des examens bactériologiques et radiologiques. Le traitement précoce de notre patient a favorisé la bonne évolution clinique et la restitution ad integrum de toutes les lésions.

La prévention des infections à Hib se base sur la vaccination et l´antibioprophylaxie. Le vaccin contre l´Hib est bien toléré et très efficace puisque plus de 90% des enfants développent des anticorps protecteurs contre l´Hib après trois doses [1]. Il est important de débuter la vaccination dès le deuxième mois car les méningites surviennent dès le troisième mois de vie. Le schéma vaccinal tunisien comporte trois injections (à 2, 3 et 6 mois). Depuis la vaccination, le portage a considérablement diminué, actuellement, les enfants plus âgés et les adultes sont plus susceptibles d'abriter le germe (porteurs asymptomatiques) et forment probablement le réservoir primaire [11]. Même si le nombre d´infections à Hib a considérablement diminué ces deux dernières décades grâce à la vaccination, un diagnostic précoce et un traitement rapide restent indispensables car le temps d´incubation pour les infections invasives est court et l´évolution de la maladie est rapide avec une létalité non négligeable et un risque élevé de séquelles neurologiques permanentes (épilepsie, retard intellectual…). L´*Haemophilus influenzae* propage par voie aérogène, par contact salivaire ou par contact avec des objets contaminés. Une antibioprophylaxie doit être mise en place rapidement chez certaines personnes de l´entourage du cas index pour éviter la propagation de la maladie.

## Conclusion

La méningoencéphalite à Hib est une pathologie rarement rencontrée, depuis la généralisation de la vaccination, mais potentiellement grave. Le tableau clinico-biologique peut être trompeur nécessitant une plus grande vigilance de la part des professionnels de santé. Tout retard du diagnostic ou du traitement peut engager le pronostic vital ou fonctionnel essentiellement neurologique. La prévention des infections à Hib se base sur la vaccination et l´antibioprophylaxie des sujets contacts de tout patient ayant une infection confirmée à Hib.
